# Serial Imaging Follow-Up for Immunoglobulin G4-Related Coronary Arteritis With Acute Coronary Syndrome

**DOI:** 10.1016/j.jaccas.2024.102561

**Published:** 2024-10-02

**Authors:** Satoshi Hata, Shingo Ota, Yasushi Ino, Masaoki Miyamoto, Yasushi Okumoto, Keizo Kimura, Atsushi Tanaka

**Affiliations:** a Department of Cardiology, Kinan Hospital, Wakayama, Japan; b Department of Cardiovascular Medicine, Wakayama Medical University, Wakayama, Japan

**Keywords:** immunoglobulin G4–related disease, intravascular ultrasonography, positron emission tomography–computed tomography, ST-segment elevated myocardial infarction

## Abstract

A 49-year-old Japanese man received a diagnosis of immunoglobulin G4–related coronary arteritis (IgG4-RCA), discovered following the detection of abdominal aorta wall thickening on computed tomography (CT). Intravascular ultrasonography (IVUS) revealed thickening of both the adventitia and the intima-media complex (IMC) in the left anterior descending (LAD) coronary artery, without significant stenosis. Corticosterone therapy was administered. On the fifth day of corticosterone therapy, the patient experienced an acute coronary syndrome secondary to LAD artery ostium occlusion, and a primary percutaneous coronary intervention was performed. After 3 months of corticosterone therapy, IVUS follow-up showed a decrease in the adventitia and IMC thickening. After 9 months of corticosterone therapy, positron emission tomography combined with CT revealed that the abnormal accumulation of fluorodeoxyglucose in the coronary arteries and abdominal aorta had disappeared. Considering the treatment process and the existing literature, there is a possibility that the adventitia and IMC deformation was induced by IgG4-RCA.

## Graphical Abstract

**Figure:**
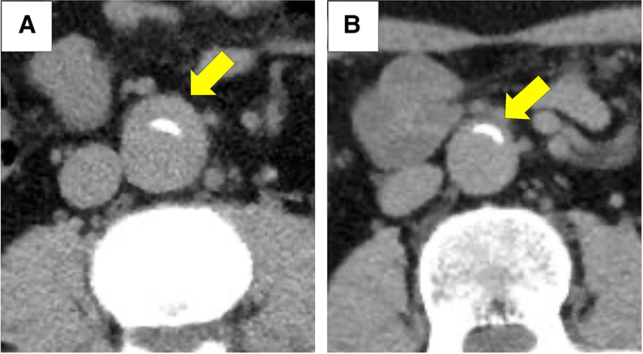

## History of Presentation

**Figure 1:**
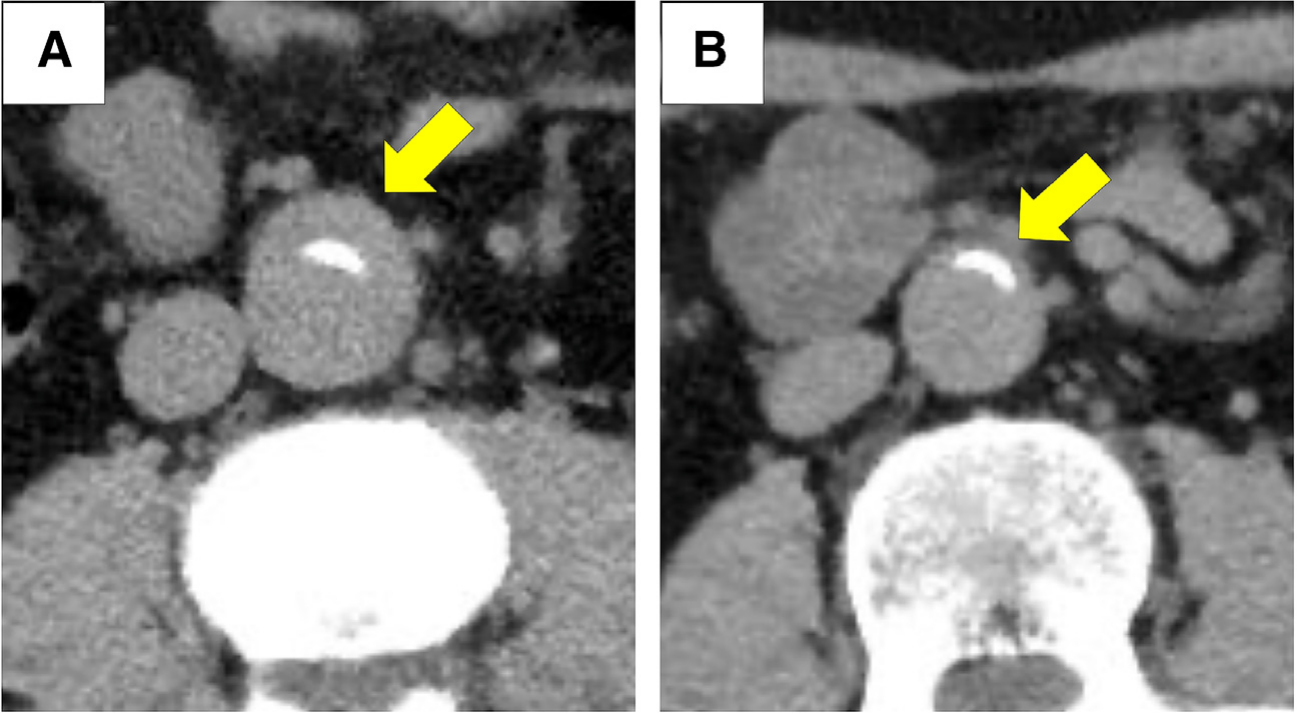
Changes in Abdominal Aorta Diameter in Computed Tomography (A) Two years after the initial visit, the diameter enlarged to 34 mm (arrow). (B) On the second month of corticosterone therapy, the wall thickening disappeared (arrow).

**Figure 2:**
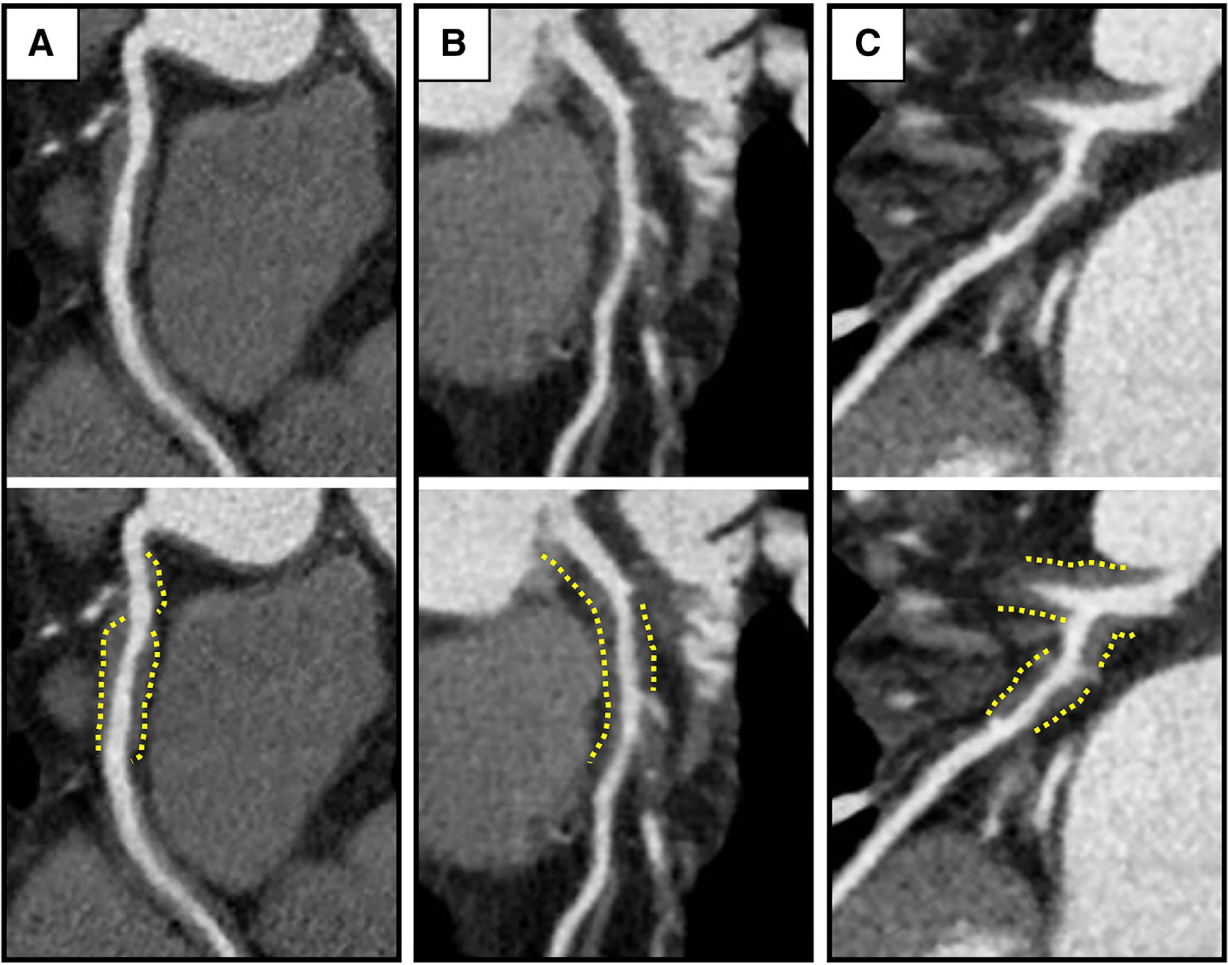
Wall Thickening of the Coronary Arteries (A) Right coronary artery. (B) Left anterior descending coronary artery. (C) Left circumflex artery. Each coronary artery has obvious artery wall thickening.

**Figure 3:**
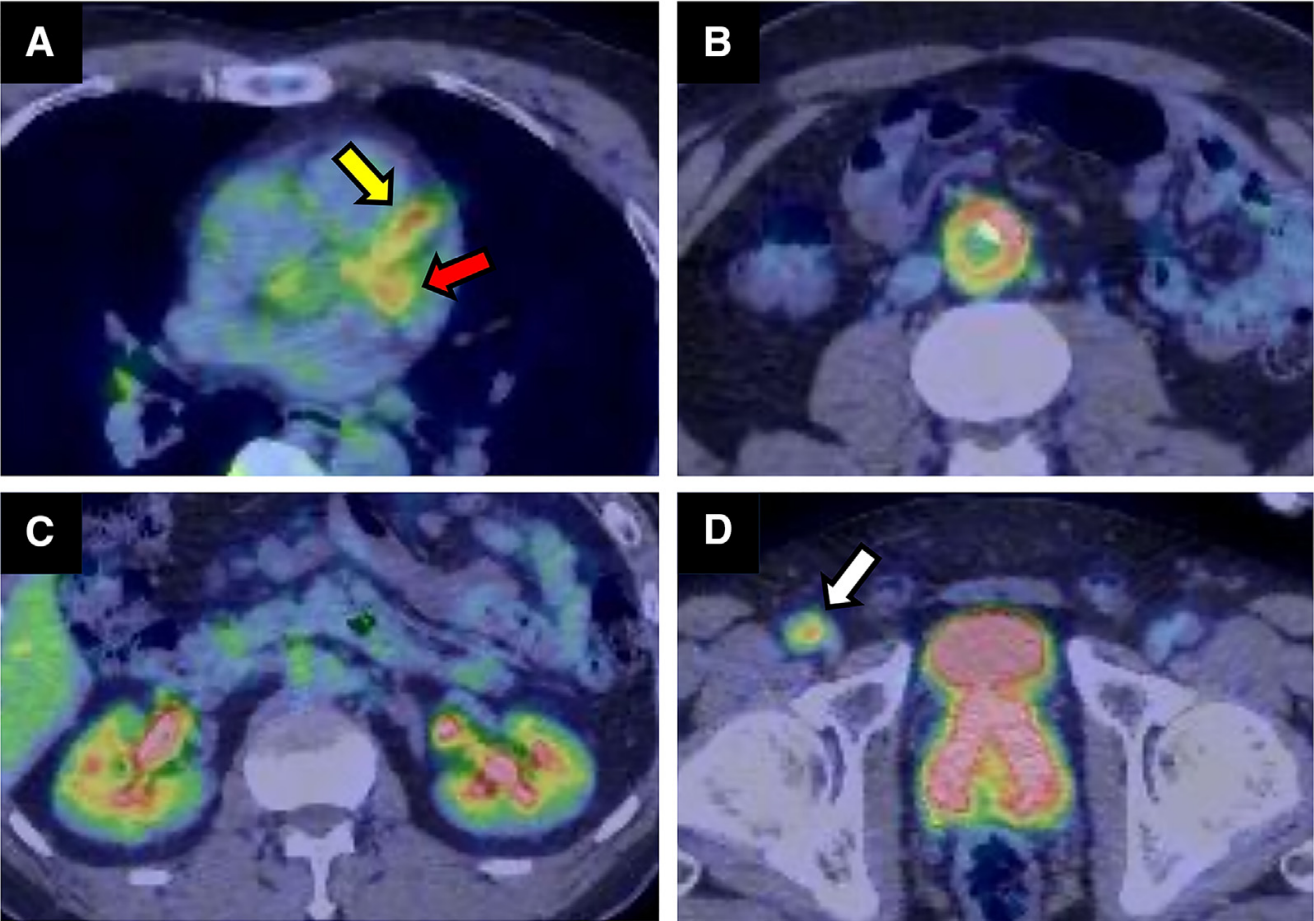
Positron Emission Tomography–Computed Tomography An abnormal accumulation of fluorodeoxyglucose was visualized in several organs. (A) Left anterior descending coronary artery (yellow arrow) and left circumflex artery (red arrow). (B) Abdominal aorta with circumferential abnormal accumulation. (C) Both kidneys, especially around the renal pelvis. (D) The prostate and the right femoral artery (white arrow).

A 49-year-old Japanese man was referred to our hospital (Kinan Hospital, Wakayama, Japan) because of asymptomatic abdominal aorta wall thickening detected during an ultrasound examination. Computed tomography (CT) revealed mild wall thickening of the abdominal aorta. After 2 years of follow-up, contrast-enhanced CT showed dilation of the abdominal aorta to 34 mm (Figures 1A and 1B); other vascular wall thickening was also detected in the coronary arteries (Figures 2A to 2C) and the right common femoral aorta. Systemic arteritis was suspected. Abnormal accumulation of fluorodeoxyglucose was found in the abdominal artery, coronary arteries, kidneys, and prostate on positron emission tomography-CT (PET-CT) (Figures 3A to 3D). Serum immunoglobin G (IgG) and IgG4 levels were elevated to 2,166 mg/dL and 1,330 mg/dL, respectively.

**Take Home Messages**

• Atypical vascular wall thickening is key to detecting systemic arteritis.
• Surveying the entire body by using multiple imaging modalities, including intravascular imaging, leads to an accurate diagnosis of arteritis.

## Past Medical History

The patient had been treated for dysuria and benign prostatic hyperplasia for several years. He also had a medical history of dyslipidemia, for which treatment with statins had been initiated.

## Differential Diagnosis

CT angiography showed systemic artery wall thickening including the coronary arteries. PET-CT showed an abnormal accumulation of fluorodeoxyglucose in these thickening systemic arteries including the coronary arteries. Serum IgG4 levels were elevated. These findings suggested IgG4-related arteritis.

## Investigations

**Figure 4:**
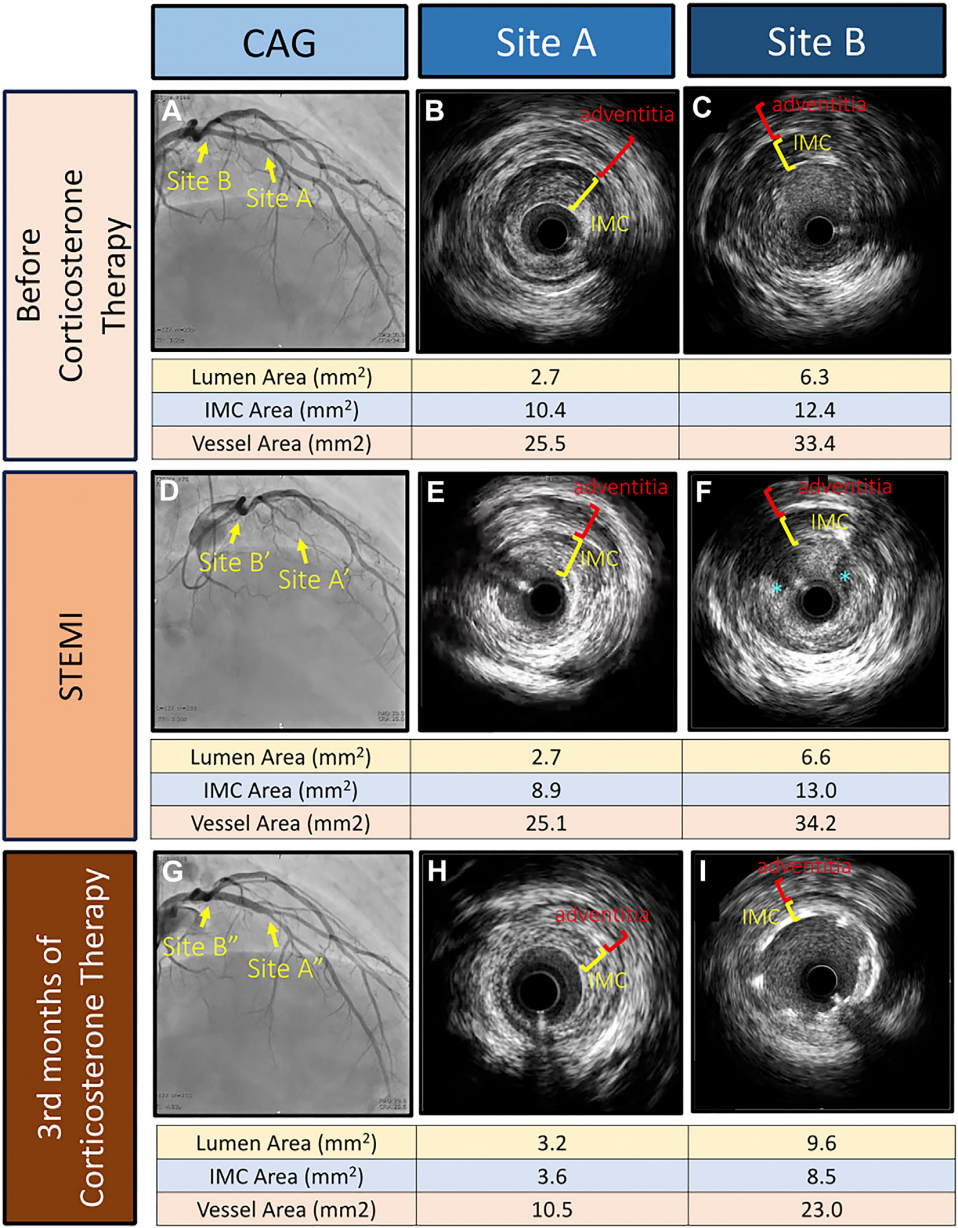
CAG and Intravascular Ultrasonography of the Left Anterior Descending Coronary Artery The most stenotic site (site A) and the culprit site in acute coronary syndrome (site B) are shown. (A) Coronary angiography (CAG) showed stenosis of the left anterior descending coronary artery. (B and C) Intravascular ultrasonography showed adventitia thickening with obvious vessel area enlargement. Intravascular ultrasonography also detected intima-media complex (IMC) thickening with nonhomogeneous low echogenicity. (D) Coronary angiography in ST-segment elevation myocardial infarction on the fifth day of steroid therapy. (E) Intravascular ultrasonography at site A. (F) Intravascular ultrasonography image at the culprit site showed intramural thrombus (asterisks). (G and I) Coronary angiography and intravascular ultrasonography on the third month of steroid therapy showed no in-stent restenosis. (H) Intravascular ultrasonography showed the decrease in the adventitia and intima-media complex thickening.

Coronary angiography (CAG) was performed because coronary arteritis was suspected. CAG revealed 50% stenosis in the proximal left anterior descending (LAD) coronary artery, referred to as site A in Figure 4A. Intravascular ultrasonography (IVUS) revealed adventitia thickening with obvious vessel area enlargement in each coronary artery. IVUS also detected intima-media complex (IMC) thickening in some regions with nonhomogeneous low echogenicity (Figures 4B and 4C). Because serum IgG4 levels were remarkably elevated, we made a diagnosis in this patient of IgG4-related arteritis involving the abdominal aorta, right common femoral artery, and coronary arteries.

## Management

**Figure 5:**
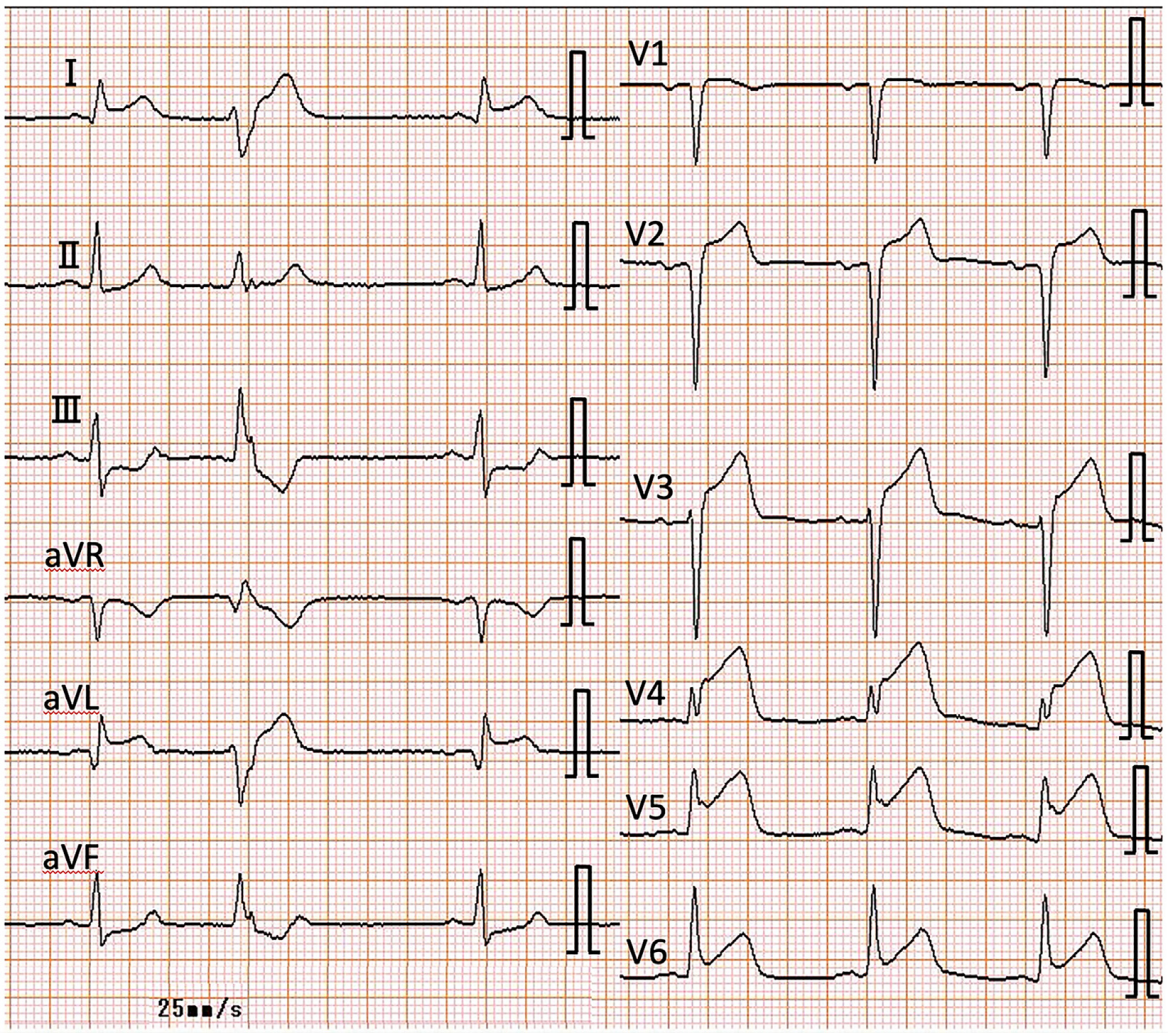
Electrocardiogram in ST-Segment Elevated Myocardial Infarction An electrocardiogram indicated ST-segment elevation in leads I, aVL, and V_2_ to V_6_.

**Figure 6:**
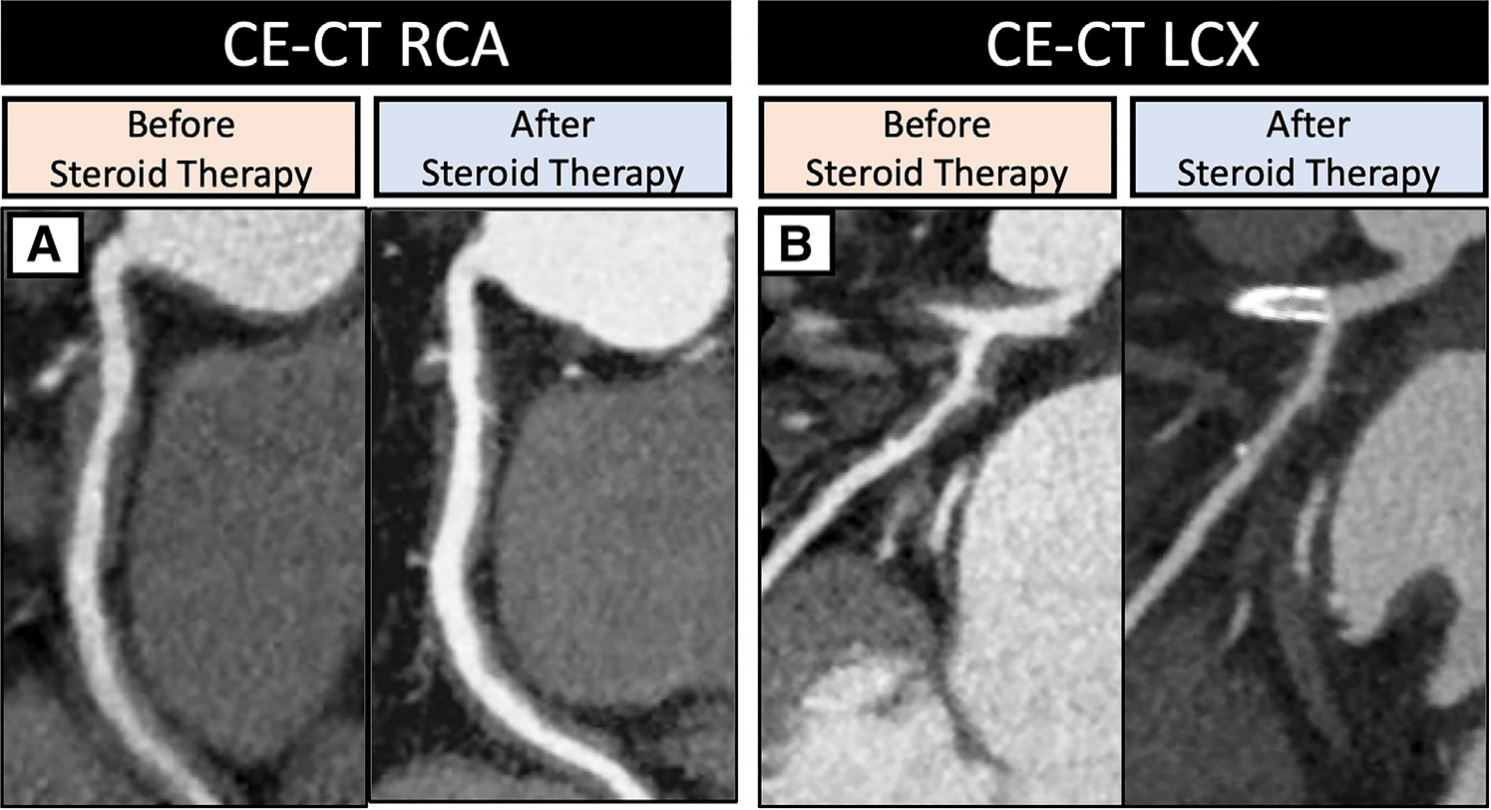
Contrast-Enhanced Computed Tomography on the Third Month of Corticosterone Therapy The wall thickening was significantly decreased in (A) the right coronary artery and (B) the left circumflex artery. CE-CT = contrast-enhanced computed tomography; LCX = left circumflex artery; RCA = right coronary artery.

On the basis of previous literature, prednisolone at 0.6 mg/kg/day was administered.^1^ On the fifth day of steroid therapy, the patient suddenly reported chest pain. An electrocardiogram indicated ST-segment elevation in leads I, aVL, and V_2_ to V_6_ and reciprocal changes in leads II, III, and aVF; the serum troponin I value was also slightly increased (Figure 5). He received a diagnosis of ST-segment elevation myocardial infarction. Emergency CAG revealed LAD artery ostium occlusion, referred to as site B, and a primary percutaneous coronary intervention was performed without any complications (Figures 4D to 4F). The prednisolone dose had been gradually reduced as initially planned. In addition, in the second month of steroid therapy, CT showed a normalized arterial wall of the abdominal aorta (Figure 1B). On the third month of steroid therapy, the prednisolone dose was reduced to 0.3 mg/kg/day, and contrast-enhanced CT follow-up showed a decrease in vessel size of the coronary arteries (Figures 6A and 6B). Moreover, IVUS follow-up showed a decrease in the adventitia IMC thickening (Figures 4G to 4I).

## Outcome and Follow-Up

**Figure 7:**
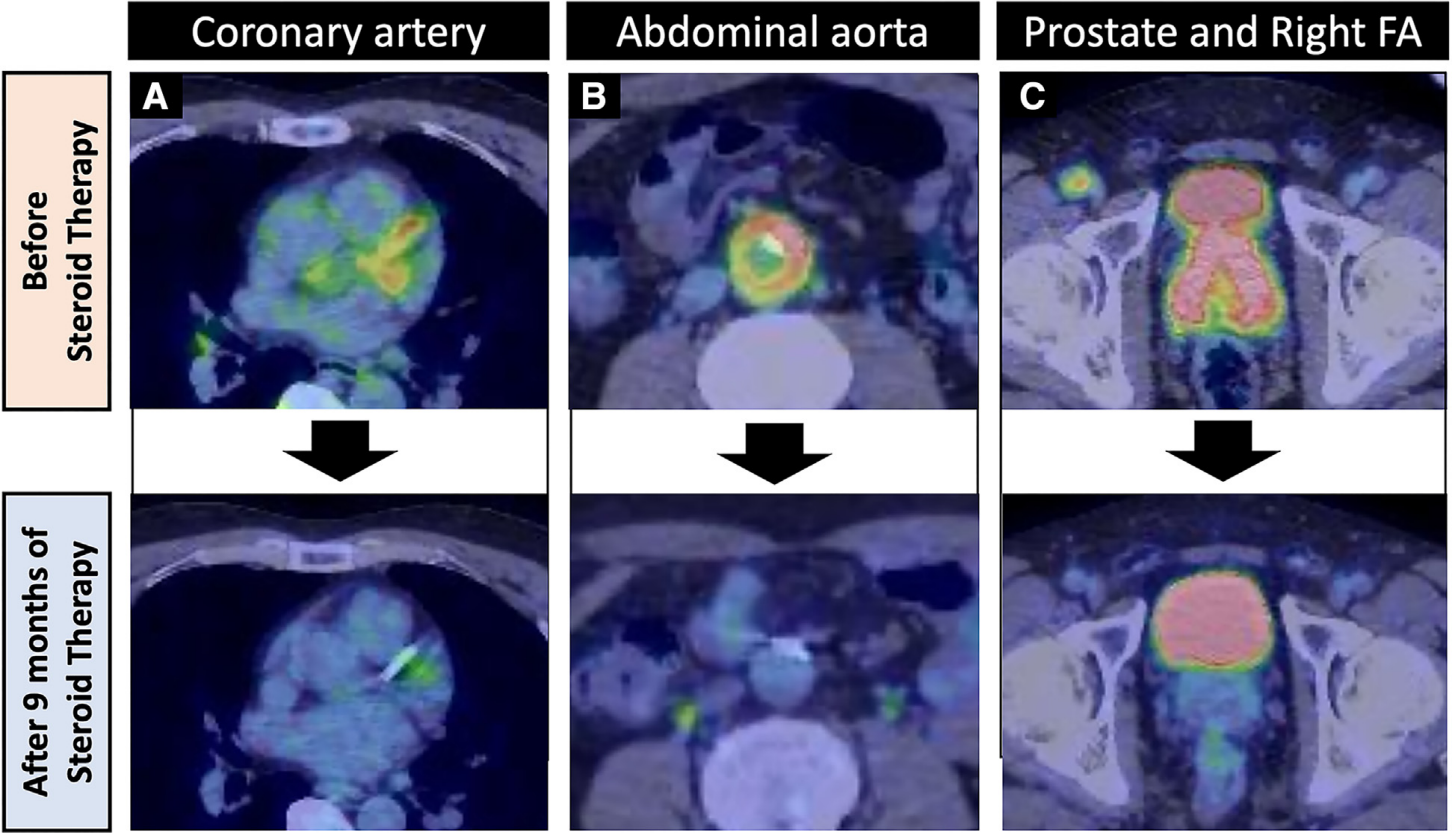
Serial Positron Emission Tomography–Computed Tomography Images An abnormal accumulation of fluorodeoxyglucose in the coronary arteries, abdominal aorta, prostate, and right femoral artery disappeared after 9 months of corticosterone therapy. (A) The left anterior descending coronary artery and left circumflex artery. (B) The abdominal aorta. (C) The prostate and the right femoral artery (FA).

On 6-month follow-up, the prednisolone dose was decreased to 0.3 mg/kg/day, and the serum IgG and IgG4 levels decreased from 2,166 mg/dL and 1,330 mg/dL to 648 mg/dL and 96 mg/dL, respectively. In addition, dysuria was also improved, and the medication for that condition was discontinued. Moreover, after 9 months of corticosterone therapy, the prednisolone dose was decreased to 0.2 mg/kg/day, and PET-CT revealed that the abnormal accumulation of fluorodeoxyglucose in the coronary arteries, abdominal aorta, prostate, and right femoral artery had disappeared (Figures 7A to 7C).

## Discussion

IgG4-related disease (IgG4-RD) is characterized by high serum IgG4 concentrations and pathologically IgG4-positive plasma cell infiltrations.^1^ The diagnosis of IgG4-RD is made on the basis of the American College of Rheumatology/European League Against Rheumatism criteria, but it is challenging because there are multiple exclusions involving clinical, serologic, radiologic, and pathologic features.^2^ IgG4-related arteritis is defined as arterial wall thickening, particularly fibrous change of the adventitia, and numerous inflammatory cell infiltrations. The most commonly affected sites in IgG4-related arteritis are the infrarenal abdominal aorta and iliac arteries and sometimes also the coronary arteries. Corticosterone therapy is the first-line medication.^1^ Symptomatic IgG4-related coronary arteritis (IgG4-RCA) mostly manifests with an ischemic event, and it may take several months or years to establish an accurate diagnosis.^3^

In this case, the decrease in adventitia and IMC thickening was observed after 3 months of corticosterone therapy by serial CT and IVUS study. To the best of our knowledge, this is the first patient with IgG4-RCA whose changes in thickening of the adventitia and IMC after corticosterone therapy could be evaluated by serial IVUS study. In patients with IgG4-RCA, histologic changes are typically observed in the adventitia.^1^ Conversely, there have been reports of an autopsy case in which IgG4-positive cells infiltrated the intima, media, and adventitia of the coronary arteries.^4^^,^^5^ Moreover, there have also been reports of IgG4-related arteritis in which IgG4-positive cells invaded not only the adventitia but also the intima and media in an abdominal aortic aneurysm with impending rupture.^6^ Therefore, we speculated that the thickening in the adventitia and the IMC was caused by IgG4-RCA in this case.

This patient experienced an acute coronary syndrome (ACS) immediately after starting corticosterone therapy. The relationship between the occurrence of ACS and IgG4-RCA has not been fully elucidated. However, there have been 2 case reports suggesting a link between acute myocardial infarction and the intimal lesions of IgG4-RCA. One case report showed that erosion with red thrombus on optical coherence tomography and yellow plaque on coronary angioscopy were observed in a patient with IgG4-RCA.^7^ Moreover, in an autopsy case of sudden death diagnosed post mortem with IgG4-RCA, pathologic examination of the coronary arteries revealed IgG4-positive plasma cells in the intima, media, and adventitia. The coronary artery was occluded by a thrombus, a finding strongly suggesting acute myocardial infarction.^4^ Compared with other intravascular imaging modalities, such as optical coherence tomography and angioscopy, a relatively low resolution of IVUS makes it difficult to evaluate the changes in the vessel wall in ACS such as plaque rupture, erosion, and infiltration of inflammatory cells accurately. Therefore, the mechanism of ACS in this case was unknown.

## Conclusions

We reported on a patient with IgG4-RCA who underwent serial multiple imaging follow-up of the coronary arteries. This patient experienced ACS immediately after starting corticosterone therapy. Serial IVUS examinations revealed the deformation of not only the adventitia but also the IMC before therapy, as well as a decrease in the adventitia and IMC area, and serial PET-CT revealed that the inflammatory changes in the coronary arteries disappeared after corticosterone therapy.

## Funding Support and Author Disclosures

This study was funded by Wakayama Medical University. The authors have reported that they have no relationships relevant to the contents of this paper to disclose.
